# Do habitat fragmentation and degradation influence the strength of fine-scale spatial genetic structure in plants? A global meta-analysis

**DOI:** 10.1093/aobpla/plad019

**Published:** 2023-05-03

**Authors:** Ara Miguel-Peñaloza, Carlos A Cultid-Medina, Jessica Pérez-Alquicira, Yessica Rico

**Affiliations:** Instituto de Ecología A.C., Red de Diversidad Biológica del Occidente Mexicano, Centro Regional del Bajío, Pátzcuaro, Michoacán 61600, Mexico; Instituto de Ecología A.C., Red de Diversidad Biológica del Occidente Mexicano, Centro Regional del Bajío, Pátzcuaro, Michoacán 61600, Mexico; CONAHCYT, Ciudad de México 03940, México; CONAHCYT, Ciudad de México 03940, México; Laboratorio Nacional de Identificación y Caracterización Vegetal, Departamento de Botánica y Zoología, Centro Universitario de Ciencias Biológicas y Agropecuarias, Universidad de Guadalajara, Zapopan, Jalisco 45200, Mexico; Instituto de Ecología A.C., Red de Diversidad Biológica del Occidente Mexicano, Centro Regional del Bajío, Pátzcuaro, Michoacán 61600, Mexico

**Keywords:** Anthropogenic habitat modification, *Sp* statistic, systematic review, plant life-history traits, weighted meta-analysis

## Abstract

As primarily sessile organisms, plants often show a non-random spatial distribution of genotypes over distance. This process known as fine-scale spatial genetic structure (FSGS) has been suggested through systematic reviews to depend on life form, mating system, and pollen and seed dispersal vectors, while there is no consensus on its behaviour due to external factors, such as anthropogenic habitat changes. By conducting a systematic review and global meta-analysis of empirical FSGS studies, we aimed to evaluate how anthropogenic habitat fragmentation and degradation influence the strength of FSGS in plant populations by means of the *Sp* statistic. Moreover, we tested how pollination and seed dispersal vectors contribute to the variation of the *Sp* statistic. We retrieved 243 FSGS studies from 1960 to 2020 of which only 65 were informative for the systematic review. Most empirical studies comprised outcrossers (84%) and trees (67%), with few herbs (23%) and scarce annual species (2%). In weighted meta-analyses for 116 plant populations (31 studies), we did not detect significant effects in the magnitude of *effect sizes* for the *Sp* statistic among undisturbed, degraded and fragmented habitats. Results showed significant effects for seed dispersal vectors, but not for pollination. Overall, we observed high variation among the *effect sizes* (not related to the goodness-of-fit of mixed models) of habitat status, pollination and seed dispersal categories, which precludes identifying biological trends on the *Sp* statistic. More empirical studies are needed that contrast multiple plant populations in disturbed versus undisturbed habitats, and by increasing the taxonomic groups, such as herbs and annual plants.

## Teaser

In our global systematic review and meta-analysis based on empirical studies, we found no evidence that habitat fragmentation and degradation influenced the strength of fine-scale spatial genetic structure across several plant species and life forms (116 plant populations in 31 studies). Due to the multiple factors affecting plant genetics, detecting one-directional trends in human-modified habitats may be challenging.

## Introduction

Anthropogenic modification of native habitats, including loss, isolation and degradation, are major drivers of biodiversity loss in terrestrial ecosystems (Fischer and Lindenmayer 2007). Plants are particularly susceptible to these habitat changes due to their sessile nature and dependency on biotic and abiotic vectors for pollen and seed dispersal (Auffret *et al*. 2017). Habitat loss and fragmentation, which modifies the area, connectivity and spatial configuration, may result in the erosion of genetic diversity and the increase of differentiation due to genetic drift and limited gene flow (Young *et al*. 1996; Aguilar *et al*. 2008, 2019). Within small and isolated fragments, the decrease in the number of reproductive individuals and the restricted dispersal of pollen and seeds can increase the spatial aggregation of related individuals, a phenomenon known as fine-scale spatial genetic structure (hereafter FSGS; Vekemans and Hardy 2004). Likewise, anthropogenic habitat degradation, which decreases habitat quality (Rykiel 1985; Fischer and Lindenmayer 2007), can change the microhabitat conditions for seedling establishment and survival (De Vere *et al*. 2009; Filazzola *et al*. 2020), influencing the strength of FSGS (Alcalá *et al*. 2015; Chiriboga-Arroyo *et al.* 2021). Such effects can be counterbalanced by the species’ life-history traits (Aguilar *et al.* 2008). Investigating the consequences of anthropogenic habitat modification on the strength of FSGS is relevant for species conservation as it can inform on inbreeding, gene dispersal, local adaptation and regeneration dynamics of plant populations (Kalisz *et al*. 2001; Jacquemyn *et al*. 2012).

Fine-scale spatial genetic structure is the result of gene dispersal, demographic and environmental factors interacting at the local scale (Heywood 1991; Vekemans and Hardy 2004; Dick *et al*. 2008). Life-history traits, such as life stage, life form, mating system and dispersal vectors can influence the strength of FSGS. For instance, stronger FSGS has been suggested in herbs and selfers relative to perennials and outcrossers (Vekemans and Hardy 2004). Also, differences in the strength of FSGS have been attributed to pollen and seed dispersal vector efficiency (Torimaru *et al*. 2007; Dick *et al*. 2008; Hoban *et al*. 2014; Gelmi-Candusso *et al*. 2017). Moreover, within the same plant population, differences can be found through life stages (Kalisz *et al.* 2001; Troupin *et al*. 2006; Kloss *et al*. 2011).

Traditionally, FSGS is characterized by autocorrelation indices, such as Moran´s I, which precludes direct comparisons between species and populations. Hence, Vekemans and Hardy (2004) proposed the *Sp* statistic, which is a synthetic measure that quantitatively characterizes the strength of FSGS in plant populations. The *Sp* statistic assumes an isotropic dispersal process and a linear decrease of pairwise relatedness (*F*_*ij*_) with the logarithm of the spatial distance separating individuals. The authors suggested, that if the latter is met, the statistic is insensitive to the sampling scheme and spatial scale (Vekemans and Hardy, 2004). The *Sp* statistic is not a standardized index, but higher values denote a strong relationship of *F*_*ij*_ over distance (Vekemans and Hardy 2004). Since its development, the *Sp* statistic has been widely employed in FSGS studies to make direct comparisons among studies, sampling sites and species (Vekemans and Hardy 2004; Dick *et al*. 2008; Gelmi-Candusso *et al*. 2017; Goncalves *et al.* 2022).

Through the *Sp* statistic, several reviews have synthesized some trends in the strength of FSGS in plants. For example, Vekemans and Hardy (2004) found that the mating system and population density influence the strength of the *Sp* statistic, which was also reported for tree species of the tropical dry forest (TDF; Goncalves *et al*. 2022). Moreover, Dick *et al*. (2008) found in temperate and tropical tree species that pollen and seed dispersal vectors influence the strength of the *Sp*. With a similar focus, Gelmi-Candusso *et al.* (2017) found among diverse plant taxa that seed disperser mobility, foraging and post-feeding behaviours of animal vectors influence the strength of the *Sp* statistic, being the species dispersed by small terrestrial animals the ones that presented the highest FSGS. Overall, available reviews suggest a relationship between life-history traits and the strength of FSGS, while little information exists on the effects of anthropogenic habitat modification, except for the recent review of Goncalves *et al.* (2022) that found no differences in the magnitude of the *Sp* statistic between tree populations of continuous and fragmented TDFs.

Studies investigating the influence of habitat fragmentation and degradation on the strength of FSGS have provided contradictory results. For example, Collevatti *et al*. (2014) found weaker FSGS for a savanna tree (*Annona crassiflora*) in a pristine habitat relative to a highly fragmented and degraded habitat. Similarly, a strong FSGS was found for seedlings of the white cedar (*Dysoxylum malabaricum*) in highly degraded and fragmented habitats (Bodare *et al*. 2017). In contrast, weak FSGS was found for the Norway spruce (*Picea abies*) in degraded habitats (Piotti *et al.* 2018), and the same was reported for adult trees of the African locust bean (*Parkia biglobosa*) in a highly fragmented landscape (Lompo *et al*., 2020).

Hence, in this study by performing weighted meta-analyses in empirical FSGS studies in plants, we aimed to answer the following questions: (i) does anthropogenic habitat modification (i.e. fragmentation and degradation) influence the strength of FSGS in plant populations in terms of the variation of the *Sp* statistic across studies? Because pollen and seed dispersal vectors are expected to influence the strength of FSGS (Vekemans and Hardy 2004), we also asked (ii) does the variation of the *Sp* statistic in undisturbed and human-modified habitats differ by the type of pollen and seed dispersal vectors? We assume that undisturbed habitats represent the ‘natural’ ecological context in which vectors persist. We expect to detect differences in the strength of the *Sp* between undisturbed and human-modified habitats. On one hand, if anthropogenic habitat modification reduces population density and gene dispersal at the local scale (e.g. Ismail *et al*. 2012; Collevatti *et al.* 2014; Bodare *et al.* 2017), we might detect stronger FSGS in highly fragmented habitats relative to undisturbed habitats. On the other hand, if anthropogenic habitat modification increases pollen and seed dispersal rates due to reduced population density (e.g. Born *et al*. 2008; Duminil *et al*. 2016) or by a random loss of related genotypes at the local scale (e.g. Parker *et al*. 2001; Chung *et al*. 2003; Yamagishi *et al.* 2007), we might expect lower FSGS in modified habitats relative to undisturbed ones. Such effects are likely to vary between biotic and abiotic pollen and seed vectors (e.g. Dick *et al*. 2008; Gelmi-Candusso *et al.* 2017). Based on a quantitative analysis of a global scope, we hope to identify general trends on the effects of anthropogenic habitat modification on the strength of FSGS in plants.

## Material and Methods

### Systematic review

We carried out a search in April 2020 on two search engines: Scopus and Web of Science using the following keywords strings: ‘fine scale’ OR ‘local scale’ AND ‘spatial genetic structure’ AND ‘plants’. The search was made starting from 1980 in Scopus and from 1960 in Web of Science. We complemented it with a backward citation search (backward snowball search) from the reviews of Vekemans and Hardy (2004), Dick *et al*. (2008) and Gelmi-Candusso *et al*. (2017).

We selected publications that met the following criteria: (i) conducted in natural habitats, (ii) conducted in undisturbed habitat or/and modified habitats where the agent of anthropogenic habitat modification is specified (e.g. logging, agriculture, mining), (iii) *Sp* statistic and (iv) significance of the *Sp* are reported (e.g. *P* < 0.05). Because our aim was to evaluate if the strength of the FSGS differs between habitat conditions (see below), we only kept studies with significant *Sp* as evidence of FSGS, in which *Sp* values can subsequently be compared among populations. For each population per study, we compiled the following data: (i) species scientific name, (ii) family, (iii) genetic marker, (iv) *Sp* statistic and significance and (v) parameters *b*_*F*_ and *F*_(1)_. The statistic was estimated as *Sp =* −*b*_*F*_/ (1 − *F*_(1)_) (Vekemans and Hardy 2004), where *b*_*F*_ is the slope from the linear regression of kinship coefficients (Loiselle *et al.* 1995) on the logarithm of the spatial distance *ln(r)*, while *F*_(1)_ is the mean kinship coefficient from the first distance class. We also recorded data of life history traits: (vi) growth form (tree, shrub and herb), (vii) mating system (mostly outcrossing, mostly selfing and mixed), (viii) reproductive system (dioecious, monoecious and hermaphrodite), (ix) life stage of the sampled population (adult, juvenile, seedling, mixed (a combination of two or three stages) and unknown), (x) pollination vectors and (xi) seed dispersal vectors. Pollination and dispersal vector information was obtained directly from the study or from other scientific publications. Owing to the lack of enough studies to assess the effect of life stage on the *Sp* statistic and to obtain comparable sampling circumstances among studies as much as possible, for those studies with information on three or two life stages, we discarded the seedlings and averaged the *Sp* statistic of adults and juveniles as we assumed that studies with unknown life-stage status could have sampled adults and juveniles. Pollination vectors were grouped into the following three categories: (i) abiotic (Ab), (ii) small animals (Bs), (iii), and (iv) mixed animals (Bmx). Seed dispersal vectors were classified into four categories: (i) abiotic short distance (Ab-s), (ii) abiotic long distance (Ab-l), (iii) mixed (Mix-l), and (iv) small terrestrial vectors (T-s) (details in Table 1).

**Table 1.** Pollination, seed dispersal and habitat status categories used in weighted meta-analyses.

ID	Category	Vectors or description
Pollination		
Ab	Abiotic	Wind
Bs	Small animals	Insects
Bmx	Mixed animals	Reptiles, bats, bats + bees
Seed dispersal		
Ab-s	Abiotic short distance	Gravity, autochory
Ab-l	Abiotic long distance	Water or wind
Mix-l	Mixed zoochory: terrestrial and flying vectors	Bats or birds Birds + primates + rodents Bats + birds + primates Elephants + primates + rodents Medium mammals + ungulates
T-s	Small terrestrial vectors	Ants or rodents
Habitat status		
U	Undisturbed	Pristine or well preserved
D	Disturbed	Agroforestry, low-intensity fires, use of fertilizers or pesticides
FL	Fragmented low	Small clearings, logging (one event), rural roads, or experimental fragmentation
FM	Fragmented medium	Large clearings, logging (many events), highways, agriculture, or farming. Only one type of agent can be present at the time.
FH	Fragmented high	Agriculture, farming, urbanization, mining, dam or channel construction. More than one type of agent should be present at the time, that is, agriculture + dam construction

In addition to the undisturbed habitat condition, we identified 13 agents of anthropogenic habitat change from the literature selected: (i) logging, (ii) experimental fragmentation, (iii) opening of clearings, (iv) agriculture, (v) farming, (vi) urbanization, (vii) mining, (viii) construction of dams or channels, (ix) rural roads or highways, (x) fires, (xi) silviculture or plantations, (xii) agroforestry and (xiii) use of fertilizers or pesticides. Then, we categorized these types into five categories (hereafter habitat status), which represented undisturbed habitat, disturbed (i.e. agents that can alter ecological systems, but that do not subdivide or reduce the habitat *per se*) and fragmented habitats (i.e. agents that subdivide a continuous area into smaller and isolated fragments). The five habitat status categories were (i) undisturbed (U), (ii) disturbed (D), (iii) low fragmented (FL), (iv) medium fragmented (FM) and (v) highly fragmented (FH) (Table 1).

## Meta-Analysis

### Effect size and moderators

We used weighted meta-analyses for testing the magnitude of the *effect size* considering three types of moderators: (i) habitat status (five categories), (ii) pollination vectors (three categories) and (iii) seed vectors (four categories). We quantified the *effect size* using the log response ratio (*lnR*), which is calculated as the natural logarithm of the response ratio with the R package *metafor* (Viechtbauer 2010):


$$\begin{array}{l} lnR\;=ln\left(\frac{m_{e}}{m_{c}}\right) \end{array}$$

where *m*_*e*_ and *m*_*c*_ are the means from the experimental (D, FL, FM and FH) and reference (U) groups. For calculating the variance, we use the following:


$$\begin{array}{l} \sigma_{lnR}^{2}=\frac{S_{e}^{2}}{n_{e}m_{e}^{2}}+\frac{S_{c}^{2}}{n_{c}m_{c}^{2}} \end{array}$$

where *n*_*c*_, *s*^*2*^_*c*_, *n*_*e*_ and *s*^*2*^_*e*_ are the sample size and sample variance for the reference and experimental groups, respectively. Due to the need for replicas, we could only use the data from publications with at least two populations of the same species for a given habitat status category. Therefore, our unit of analysis was a group of populations from the same study and species under one of the five habitat statuses. For each unit, we calculated the mean ($\bar{x}$), standard deviation and sample size (*n*). We used the *lnR* because it has been identified as an *effect size* measure that is robust to the natural variability of ecological data and is suitable for small sample sizes (Lajeunesse and Forbes 2003). The robust statistics of the *lnR* allow for conducting a meta-analysis of the global scope for ecological and evolutionary data, in which a portion of the studies may not have paired data of experimental and reference groups, or may not have a reference group (e.g. Romero *et al*. 2014; Brustolin *et al*. 2018; Méndez-Rojas *et al*. 2021). Because most studies did not have an undisturbed habitat condition (U), we used the mean from all U populations for the reference group. This approximation is valid as our meta-analyses will focus on evaluating if the extent of the variation of the *Sp* across studies is explained by the covariates (see below) and how the response differs among studies (Gurevitch and Nakagawa 2015).

### Effect models and publication bias

We fitted mixed-effect models to assess whether the variation in *effect size*s of the *Sp* statistic differs among the categories for habitat status, pollination and seed dispersal vectors. For this, we used the *Q*-statistic to evaluate whether the heterogeneity of each study’s *effect size* (*QM*) can be explained by the moderator and whether the variability of the observed *effect sizes* is larger than it would be expected based on sampling variability alone (i.e. residual heterogeneity, *QE*). If *QE* is not significant (*P >* 0.05), the variability observed among *effect sizes* can be explained by the moderator, and if *QM* is not significant (*P* > 0.05), the variability observed among *effect sizes* cannot be explained by the moderator. Also, we calculated the *I*^2^ statistic, which determines the ratio of the total variance explained by the between-study variance (Gurevitch and Nakagawa 2015). We used funnel plots to visualize the relationship between *effect size* and the precision for each model, and *QQ* plots to identify any outliers and biases in the studies. For each response variable, we evaluated publication bias with the Egger test (Egger *et al*. 1997) as suggested in Gurevitch and Nakagawa (2015).

Lastly, because previous studies have found evidence of the influence of mating system and life form on the strength of the FSGS (Vekemans and Hardy 2004; Gelmi-Candusso *et al.* 2017), we repeated the meta-analyses only for outcrossing species to evaluate if the mating system could have masked any effect. Outcrossing species represented 88% of the data (*n* = 24 studies). Additionally, to eliminate any bias due to the mating system and life form, we repeated the analysis only for outcrossing tree species, which represented almost half of the data (45%, *n* = 15 studies).

## Results

### Systematic review

**Figure 1.**
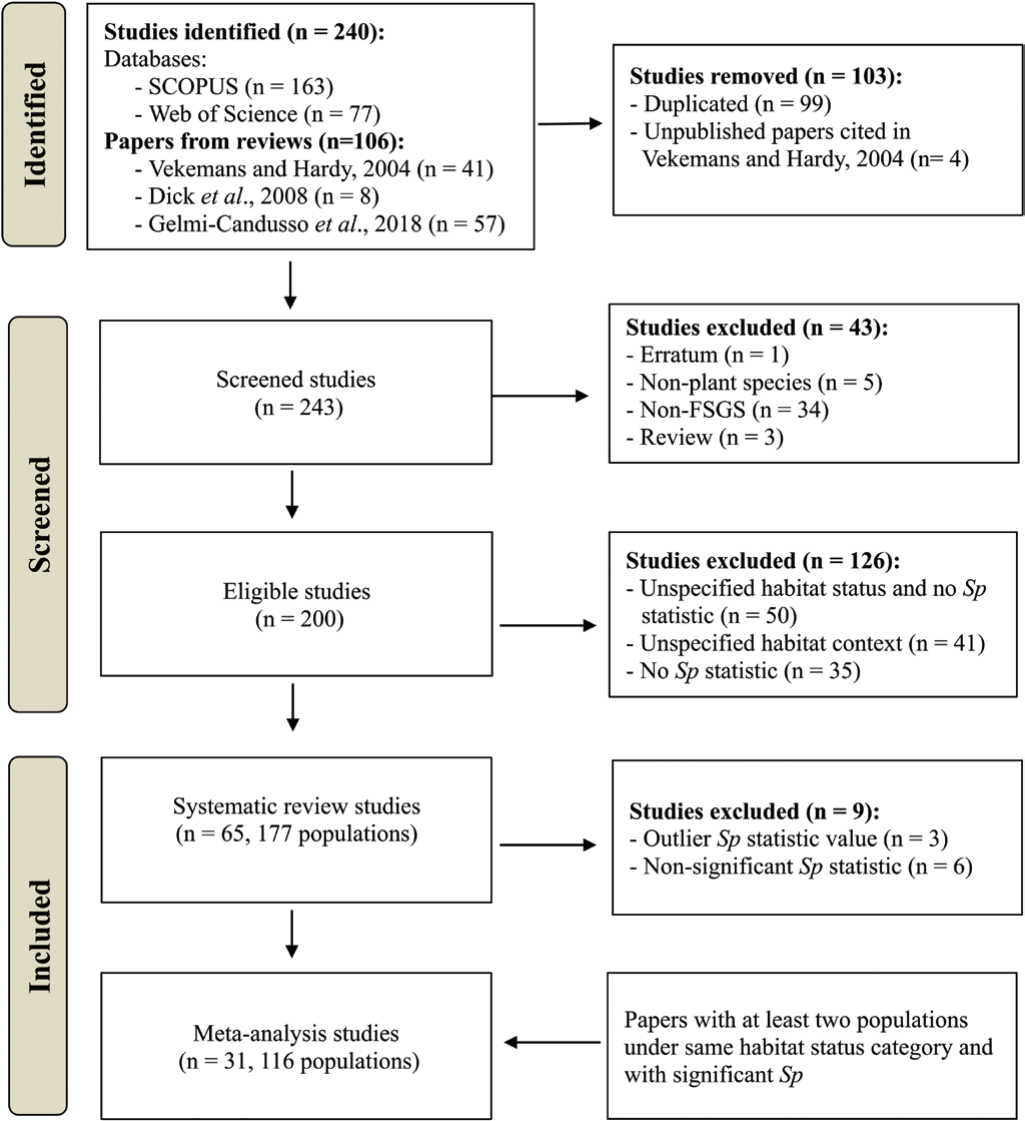
Flowchart of the data compilation for the field synopsis, systematic review and weighted meta-analysis.

Our database search retrieved 343 published studies. After an initial screening, we identified 243 non-duplicated studies, of which 40 did not meet any of the selection criteria and thus were discarded, including three FSGS reviews, for a total of 200 eligible studies. Of these 200 studies, only 74 provided information about the habitat status (i.e. the agent of anthropogenic habitat change), and the *Sp* statistic. We removed six studies with non-significant FSGS and three studies with outlier *Sp* values (ranging from 0.728 to 1.056), and thus we included only 65 published papers for the systematic review (Fig. 1, **see****Supporting Information—Table S1**). The number of publications slightly varied between years, being 2015 and 2017 the years with the most published papers (see **Supporting Information—Fig. S1**).

From the 65 publications, we compiled data on 177 plant populations. These 65 studies comprised 37 families and 73 species, with Fabaceae, Fagaceae, Rosaceae and Arecaceae the most frequent families among studies (>4 studies). The rest of the families were mostly evaluated in single studies (70.3 %; see **Supporting Information—Fig. S2**). The most common genetic marker were microsatellites (70.7 %), followed by amplified fragment length polymorphisms (12.3 %) and allozymes (9.2%), while single nucleotide polymorphisms (SNPs) were scarcely represented (1.5 %). Most species were outcrossers (87.7 %), while for the reproductive system, most were monoecious (38.4 %) and hermaphrodites (37.5 %), with the rest being dioecious (24.1 %). Tree species (67.2 % of the total population) were over-represented relative to herbs (23.2 %), while annual species were almost lacking (2 %). Most studies evaluated just one species under one habitat status category (61.5 %), followed by studies for one species under two habitat status categories (24.6%). Only 36.9 % of the 65 studies explicitly evaluated the effect of anthropogenic habitat change on the *Sp* statistic. The most represented habitat status categories were FH (23.7 % populations), then U and D (22.0 % and 22.5 %, respectively), and FL showing the lowest representation (13.6%). Most populations had either insects or wind as pollination vectors (62.7 % and 28.8 %, respectively). Finally, for seed dispersal, the large majority had an abiotic vector, either short-distance (33.3%) or long-distance (22%) dispersal. The least common seed vectors were small terrestrial animals (9%).

### Meta-analysis

For the meta-analyses, only 31 from the 65 studies met the inclusion criteria of at least two populations in one of the five habitat status categories, which resulted in 116 populations (Fig. 1). Of these 116 populations, just 6 used SNPs as genetic markers, while the rest used microsatellites.

**Figure 2.**
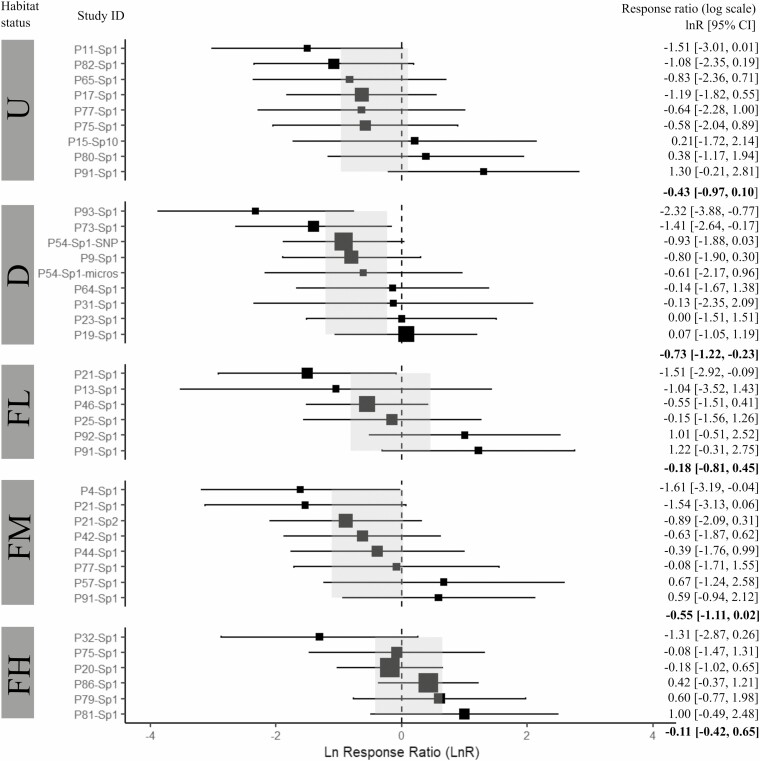
Forest plot of the mixed-effect model for habitat status. Estimated *effect sizes* (black squares) with 95% confidence intervals (CI, horizontal bars) of the *Sp* statistic for each analysed study (Study ID). The size of the black squares is proportional to the number of populations per study (minimum size = 2 populations). The dotted vertical line represents the *lnR* = 0 of no global effect of the fitted mixed model. Values in bold and light grey rectangles in the forest plot denote the mean estimate of *lnR* [95% CI] for each habitat status category. Categories: U = Undisturbed, D = Disturbance, FL = Fragmented low, FM = Fragmented medium, and FH = Fragmented high.

**Figure 3.**
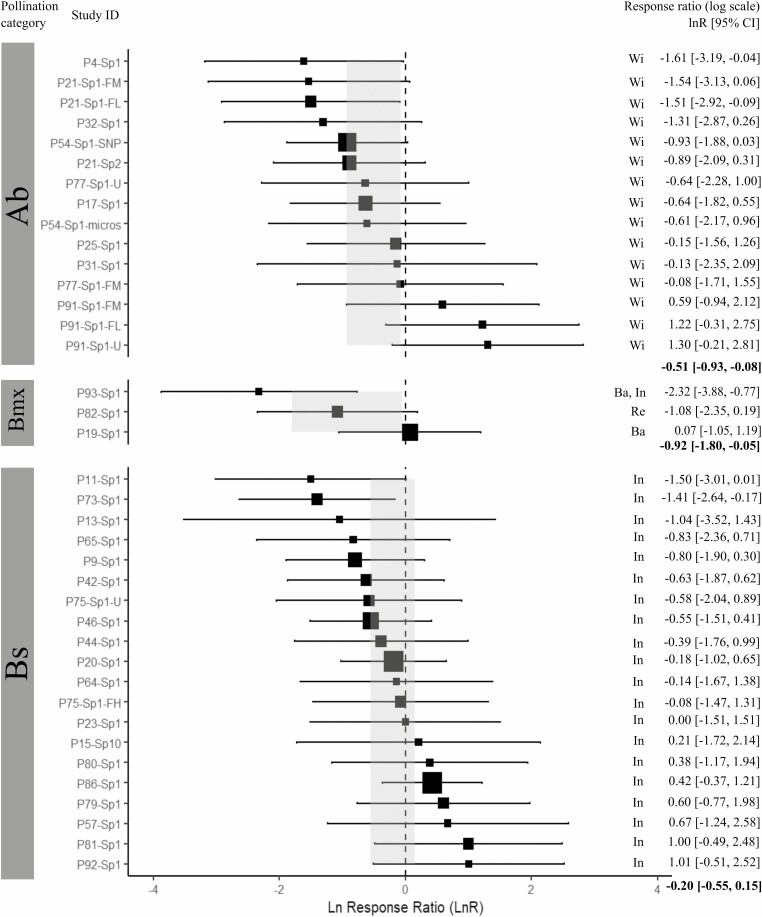
Forest plot of the mixed-effect model for pollination vectors. Estimated *effect sizes* (black squares) with 95% confidence intervals (CI, horizontal bars) of the *Sp* statistic for each analysed study (Study ID). The size of the black squares is proportional to the number of populations per study (minimum size = 2 populations). The dotted vertical line represents the *lnR* = 0 of no global effect of the fitted mixed model. Values in bold and light grey rectangles in the forest plot denote the mean estimate of *lnR* [95%CI] for each habitat status category. Categories: Ab = Abiotic; Bmx = Mixed animals; Bs = Small animals. Pollination vectors subcategories: Ba = Bats, In = Insects, Re = Reptiles, and Wi = Wind.

**Figure 4.**
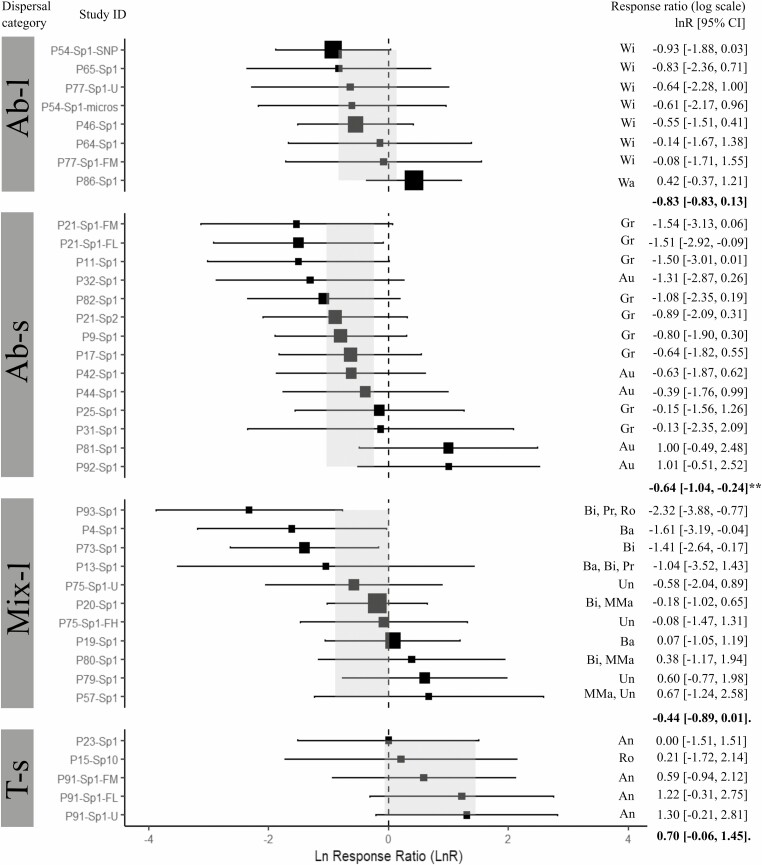
Forest plot of the mixed-effect model for seed dispersal vectors. Estimated *effect sizes* (black squares) with 95% confidence intervals (CI, horizontal bars) of the *Sp* statistic for each analysed study (Study ID). The size of the black squares is proportional to the number of populations per study (minimum size = 2 populations). The dotted vertical line represents the *lnR* = 0 of no global effect of the fitted mixed model. Values in bold and light grey rectangles in the forest plot denote the mean estimate of *lnR* [95% CI] for each habitat status category. Categories: Ab-l = abiotic long distance; Ab-s = abiotic short distance, Mix-l = Mixed abiotic and biotic vectors and T-s = small terrestrial vectors. Seed dispersal vectors subcategories: An = ants, Au = autochory, Ba = bats, Bi = birds, MMa = medium size mammals, Gr = Gravity, Pr = Primates, Ro = Rodents, Un = Ungulates, Wa = Water and Wi = Wind. *P*-values: 0 ‘***’, 0.001 ‘**’, 0.01‘*’, 0.05‘’., 0.1 ‘’.

The *Q*-statistic showed that for the three fitted models with the complete dataset, the residual heterogeneity was not statistically significant (*QE P* > 0.05), and thus the observed variability among *effect sizes* can be explained by the categories of each of the moderators (see **Supporting Information—Table S2**). However, the fixed-effect model of habitat status did not detect a significant effect in the variation of the *Sp* statistic among the five habitat status categories (*QM* = 42.7, *I*^2^ = 17.2 %, *R*^2^ = 39.1 %, *P* = 0.12; Fig. 2). For pollination vectors, the meta-analyses showed no significant effect among categories (*QM* = 2.86, *I*^2^ = 25.06 %, *R*^2^ = 3.93 %, *P* = 0.239; Fig. 3). For seed vectors, results showed a significant effect in the variation of the *Sp* statistic among the four dispersal categories (*QM* = 9.51, *I*^2^ = 15.68 %, *R*^2^ = 46.21 %, *P* = 0.023), where the category of short-distance abiotic vectors (Ab-s) was significantly different from zero (Fig. 4). The funnel and *QQ* plots of the three fitted models showed high precision (see Supporting Information—Figs. S3–S5), while the Egger test showed no publication bias (habitat status: *Z* = 0.681, *P* = 0.50; pollination vectors: *Z* = 0.179, *P* = 0.86, and seed vectors: *Z* = −-0.65, *P* = 0.51).

The meta-analysis for outcrossing species only showed similar results as with the complete dataset. The *Q*-statistic showed that the residual heterogeneity was not statistically significant (*QE*, *P* > 0.05) for any of the moderators. For habitat status, we did not find a significant effect in the variation of the *Sp* statistic (*QM* = 2.38, *I*^2^ = 63.8 %, *R*^2^ = 0 %, *P* = 0.66), and neither for pollination (*QM* = 1.626, *I*^2^ = 29.04 %, *R*^2^ = 0 %, *p* = 0.443). In contrast, seed dispersal results showed a significant effect among dispersal categories (*QM* = 13.29, *I*^2^ = 1.99 %, *R*^2^ = 94.3%, *P* = 0.003), where the *lnR* (IC 95 %) of short-distance abiotic vectors (Ab-s) and mixed zoochory (Mix-l) were significantly different from zero (see Supporting Information—Figs. S6–S8). The funnel and *QQ* plots showed moderate to high precision (see Supporting Information—Figs. S9–S11), while the Egger test showed no publication bias for any of the models (habitat status: *Z* = 0.048, *P* = 0.96; pollination vectors: *Z* = −0.304, *P* = 0.76, and seed vectors: *Z* = −0.918, *P* = 0.36). Lastly, results for outcrossing tree species showed significant residual heterogeneity for the three fitted models (*QE*, *P* < 0.05; see Supporting Information—Figs. S12–S14), and thus the observed variability in *effect sizes* of the *Sp* statistic cannot be explained by the categories of each moderator, which was likely due to small sample size. However, by looking at the forest plots, we observed a similar behaviour in the variation of the *Sp* relative to the complete and outcrossing datasets (see Supporting Information—Figs. S15–S17).

## Discussion

Understanding the genetic consequences of anthropogenic habitat fragmentation and degradation on plant populations has been a prolific area of research for the last decades. Systematic reviews in plant genetic studies in fragmented and degraded habitats have focused on synthesizing the negative effects on genetic diversity parameters, such as expected heterozygosity, allelic richness and inbreeding coefficients (e.g. Aguilar *et al.* 2008; Vranckx *et al*. 2012; González *et al*. 2020), but less attention has been given to other population parameters related to genetic structure, such as spatial patterns of fine-scale genetic structure. Previous systematic reviews have suggested that the strength of the fine-scale genetic structure in terms of the *Sp* statistic is influenced by pollination and seed dispersal vectors (Dick *et al.* 2008; Gelmi-Candusso *et al.* 2017), population density and mating system (Vekemans and Hardy 2004; Goncalves *et al.* 2022). In this study by conducting a systematic review, we aimed to evaluate how anthropogenic habitat modifications influence the magnitude of the *Sp* statistic in plant populations. Our meta-analyses showed no significant differences in the variation of the *Sp* statistic among undisturbed, degraded and fragmented habitats. This result remained unchanged when considering only outcrossing mating species. This point is relevant as a meta-analysis in plants have suggested that outcrossing species are the most sensitive to the negative effects of habitat fragmentation (Aguilar *et al.* 2008, 2019), and thus we might have expected to detect such effects by considering only outcrossing species. Our review is the first one to evaluate the variation in *effect sizes* of the *Sp* statistic through weighted meta-analyses, which had the virtue to assess if the heterogeneity observed in *effect sizes* are related or not to the categories of an explanatory factor, considering that the precision might differ across studies (Gurevitch and Nakagawa 2015).

Moreover, because pollination and seed dispersal vectors are expected to influence the strength of FSGS (Vekemans and Hardy 2004), we also analysed if pollen and seed vectors played a role in the variation of the *Sp* statistic among undisturbed and human-modified habitats. Our results from meta-analyses showed significant effects due to seed dispersal vectors, but not to pollination. Specifically, we observed that species with gravity and autochory dispersal showed the lowest (negative) estimated mean *lnR* (IC 95 %) relative to the other dispersal categories, which implies that plants with abiotic short-distance dispersal vectors showed lower FSGS. Results for outcrossing species only, in addition, showed significant effects related to mixed zoochory (terrestrial and flying vectors), which was the second category after Ab-s with the lowest estimated mean *lnR* (IC 95 %). Our results were consistent with trends observed by Goncalves *et al*. (2022), in which anemochory and autochory species had lower mean *Sp* values relative to animal-dispersed species (birds, bats, and rodents). However, our results differ from the trends shown by Vekemans and Hardy (2004) and Dick *et al.* (2008), which showed that plants dispersed by gravity have higher *Sp* than animal-dispersed species. Regarding pollination our results were consistent with those reported by Vekemans and Hardy (2004) and Goncalves *et al*. (2022), but different from those reported by Dick *et al*. (2008), who found that animal-pollinated temperate trees had higher FSGS than wind-pollinated trees. Moreover, Hardy *et al.* (2006) suggested that restricted seed dispersal can indirectly increase the contribution of seeds to FSGS as compared to pollen by increasing spatial aggregation of flowering plants, which in turn increases the probability of biparental inbreeding. Differences among studies may be related to the complexity of categorizing pollen and seed dispersal vectors, in particular for species with multiple vectors of which biological information is incomplete or unknown. In our case, the mixed-l category comprised a large variety of animal vectors, which may differ in dispersal, feeding behaviour and habitat preferences. Moreover, from the systematic reviews by Vekemans and Hardy (2004), Dick *et al*. (2008) and Gelmi-Candusso *et al.* (2017), our final database shared only 9.8 %, 37.5 % and 42.1 % of the references therein, respectively, because of the criteria needed (at least two populations per condition) to select populations for inclusion in our meta-analyses. Although this inclusion criterion may reduce the information, it strengthens the *effect sizes* estimate.

However, by looking at the forest plots of the three moderators tested and for the three datasets, it was evident that high variation within and between categories is present in the *effect size*s of the *Sp* across studies (i.e. *lnR* [IC 95 %]), and irrespective to habitat status, pollination and seed dispersal vectors. Such high variation among the *Sp* values precludes identifying clear biological trends on the strength of FSGS in plant populations as we expected. Thus, we argue that our findings might be influenced by four reasons: (i) the number of studies included in the meta-analyses was insufficient to detect any informative variation trend, (ii) the *Sp* statistic might be sensitive to spatial sampling schemes, (iii) habitat fragmentation and degradation influence FSGS in plant populations in different directions challenging to detect any effect as a one-directional trend, and (iv) time-lag effects of anthropogenic habitat changes on FSGS in plant populations. The following discusses in more detail each of these reasons.

A reduced number of studies (i) to reach conclusive results is a common scenario in many meta-analyses (Schlaepfer *et al.* 2018; González *et al.* 2020). It has been suggested that the minimum number of studies should be in the range of 20–30 (Koricheva *et al.* 2013). Our sample size was within the minimum range suggested (*n* = 116 populations in 31 studies), which should be enough to at least observe major trends. It is important to acknowledge that we could not exclusively include studies that compared FSGS in disturbed versus undisturbed sites for the same species under similar sampling schemes. Very few studies meet this condition (*n* = 4). However, the lack of significant effects cannot be attributed to a lack of statistical power in our weighted meta-analyses as we had high precision and no publication bias. The relatively low values of the *I*^2^ statistic suggested that the mixed models accounted large part of the variation of the *Sp* statistic (Gurevitch and Nakagawa 2015). We also confirmed the lack of significant effects by exploring other traditional non-parametric (Kruskal–Wallis) and parametric statistical tests (ANOVA, see **Supporting Information—Table S3**).

Regarding the (ii) *Sp* statistic influenced by spatial sampling schemes, Vekemans and Hardy (2004) in their description of the *Sp* statistic, suggested that it was insensitive to the sampling scheme and spatial scale only if the kinship coefficient decreases linearly with the logarithm of the spatial distance. If this condition is not met, the *Sp* statistic will depend on the distance range of the sampling scheme. Vekemans and Hardy (2004) recognized the challenge to assess this assumption, which is not specified in empirical FSGS studies. Recently, Hein *et al.* (2021) demonstrated two spatial metrics based on simulated data, MEMgene adjusted *R*^2^ and multivariate Moran’s *I*, their sensitivity due to demographic history, number of individuals sampled and sampling scheme. Hein *et al.* (2021) concluded that the strength of FSGS cannot be compared using these two metrics and that comparisons among studies and species are not precise and thus not warranted. Although the *Sp* statistic has been rendered insensitive to the spatial sampling scheme, this assumption has not been formally tested through computer simulations and contrasted with empirical data. Specifically, we found a large variation in spatial sampling schemes across empirical studies, which varied from intensive (47.5%), systematic (36.2%) and randomized sampling (16.4%), and with variations between life stages (3.4%).

The (iii) habitat loss, fragmentation and other disturbances are important mechanisms that can affect seed dispersal and pollen flow in multiple ways (Aguilar *et al.* 2008; Fontúrbel *et al.* 2015; González *et al.* 2020). High or weak FSGS has been observed in plant populations in human-modified habitats (e.g. Collevatti *et al.* 2014; Bodare *et al.* 2017; Lompo *et al.* 2020), which may challenge the detection of significant one-directional trends through a meta-analysis, and when other confounding factors cannot be accounted for among empirical studies (e.g. plant density, demographic history, the time elapsed since the fragmentation). For instance, many of the selected studies did not include information on population density (59.9%), which is another intrinsic factor expected to influence the strength of FSGS (Vekemans and Hardy 2004).

Time-lag effects on genetic diversity caused by anthropogenic habitat changes (iv) is another reason that seems plausible for explaining the results from our meta-analyses, as most studies were for long-lived species such as trees, while only one study corresponded to an annual forb. Vranckx *et al*. (2012), in their meta-analysis of woody species, reported that habitat fragmentation had a stronger impact on genetic diversity for the progeny relative to the adults. Moreover, studies that have compared FSGS between life stages have found that young individuals show stronger FSGS compared to adults in tree species (Browne *et al*. 2015; Bodare *et al*. 2017).

### Recommendations

We found that a large number of studies (45 %) did not include information on the habitat status of the studied populations. Thus, to better understand how FSGS vary in plant populations, it is advisable that empirical studies provide relevant information on habitat conditions, such as if there is evidence of anthropogenic habitat fragmentation and degradation. Moreover, it would be ideal to provide information on the timespan since the anthropogenic habitat modification. This information is key for understanding if plant populations experience FSGS time-lag effects and if this depends on life-history traits (Bodare *et al.* 2017). Our current data did not allow us to evaluate this possibility as very few studies provided information on the years that elapsed since the anthropogenic habitat changes. Testing any effects on life stages was not possible either, as very few studies have compared the strength of FSGS across life stages, while most of them did not specify the age category of the sampled individuals. Studies using direct gene flow estimations such as parentage analysis can reveal in more detail the current conditions of gene dispersal and if time-lag effects are present in the overall population. Additionally, if the information is known, we suggest the inclusion of data from pollen and seed dispersal vectors on the studied populations, as for the same species, pollen and seed vectors can change depending on the habitat.

More empirical studies are needed on forbs and annual species as trees are over-represented in most population genetic studies (Rico 2019; González *et al.* 2020). Lastly, to further understand the conditions under which FSGS may occur or not in human-modified landscapes, more research is required on plant population dynamics, including investigating the microhabitat conditions that promote seedling establishment and growth, as well as the variance in reproductive success.

## Conclusions

Our meta-analyses did not have enough evidence to detect significant effects of habitat fragmentation and degradation on the strength of FSGS in plant populations. According to the systematic and global meta-analysis, detecting such effects in human-modified habitats may be challenging because of the multiple factors influencing plant genetics. More empirical and standardized (i.e. detailed habitat information, information on dispersal vectors and population dynamics) studies are needed that contrast multiple plant populations in disturbed versus undisturbed habitats, and by increasing the number of taxonomic groups, such as for herbs and annual plants.

## Supporting information

The following additional information is available in the online version of this article—

**Table S1.** The dataset used for the systematic review (65 studies) and meta-analyses for the complete set (31 studies), outcrossers only and outcrossing trees.

**Table S2.** Statistics from meta-analyses with the complete, outcrossers and outcrossing tree species.

**Table S3.** Statistics from ANOVA and Kruskal–Wallis tests for the *Sp* values in 177 plant populations under five habitat status categories.

**Figure S1.** Publications on significant *Sp* statistic (or parameters) and specified habitat status.

**Figure S2.** Number of publications with significant *Sp* statistic and specified habitat status per taxonomic family.

**Figure S3.** Funnel plot and *QQplot* of the *Sp* statistic for the mixed-effect model meta-analysis with habitat status as moderator for the complete dataset.

**Figure S4.** Funnel plot and *QQplot* of the *Sp* statistic for the mixed-effect model meta-analysis with pollination category as moderator for the complete dataset.

**Figure S5.** Funnel plot and *QQplot* of the *Sp* statistic for the mixed-effect model meta-analysis with seed dispersal category as moderator for the complete dataset.

**Figure S6.** Forest plot of the mixed-effect model for habitat status of outcrossing species only.

**Figure S7.** Forest plot of the mixed-effect model for pollination vectors of outcrossing species only.

**Figure S8.** Forest plot of the mixed-effect model for seed dispersal vectors of outcrossing species only.

**Figure S9.** Funnel plot and *QQplot* of the *Sp* statistic for the mixed-effect model meta-analysis with habitat status as moderator for outcrossing species.

**Figure S10.** Funnel plot and *QQplot* of the *Sp* statistic for the mixed-effect model meta-analysis with pollination as moderator for outcrossing species.

**Figure S11.** Funnel plot and *QQplot* of the *Sp* statistic for the mixed-effect model meta-analysis with seed dispersal as moderator for outcrossing species.

**Figure S12.** Funnel plot and *QQplot* of the *Sp* statistic for the mixed-effect model meta-analysis with habitat status as moderator for outcrossing tree species.

**Figure S13.** Funnel plot and *QQplot* of the *Sp* statistic for the mixed-effect model meta-analysis with pollination as moderator for outcrossing tree species.

**Figure S14.** Funnel plot and *QQplot* of the *Sp* statistic for the mixed-effect model meta-analysis with seed dispersal as moderator for outcrossing tree species.

**Figure S15**. Forest plot of the mixed-effect model for habitat status for outcrossing trees.

**Figure S16.** Forest plot of the mixed-effect model for pollination vectors for outcrossing trees.

**Figure S17.** Forest plot of the mixed-effect model for seed dispersal vectors for outcrossing trees.

plad019_suppl_Supplementary_Figure_S1

plad019_suppl_Supplementary_Figure_S2

plad019_suppl_Supplementary_Figure_S3

plad019_suppl_Supplementary_Figure_S4

plad019_suppl_Supplementary_Figure_S5

plad019_suppl_Supplementary_Figure_S6

plad019_suppl_Supplementary_Figure_S7

plad019_suppl_Supplementary_Figure_S8

plad019_suppl_Supplementary_Figure_S9

plad019_suppl_Supplementary_Figure_S10

plad019_suppl_Supplementary_Figure_S11

plad019_suppl_Supplementary_Figure_S12

plad019_suppl_Supplementary_Figure_S13

plad019_suppl_Supplementary_Figure_S14

plad019_suppl_Supplementary_Figure_S15

plad019_suppl_Supplementary_Figure_S16

plad019_suppl_Supplementary_Figure_S17

plad019_suppl_Supplementary_Table_S1

plad019_suppl_Supplementary_Table_S2

plad019_suppl_Supplementary_Table_S3

## Data Availability

The database used for the systematic review and meta-analysis is provided as Supporting Information Table S1.

## Notes

**Evolution and Diversity. Chief Editor:** Jeremy Beaulieu
