# Supplementary figures and images for: Do habitat fragmentation and degradation influence the strength of fine-scale spatial genetic structure in plants? A global meta-analysis
Source: AoB Plants. 2023 May 3;15(3):plad019. doi: 10.1093/aobpla/plad019 (PMC10198778; doi:10.1093/aobpla/plad019)

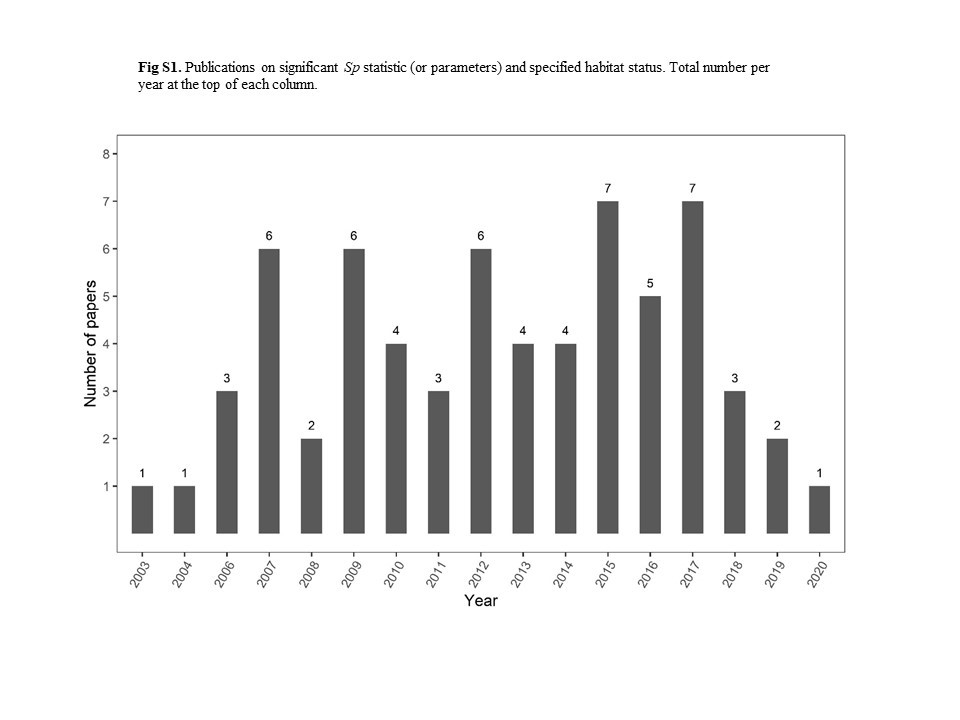

Supplement: plad019_suppl_Supplementary_Figure_S1 [file plad019_suppl_supplementary_figure_s1.jpeg]

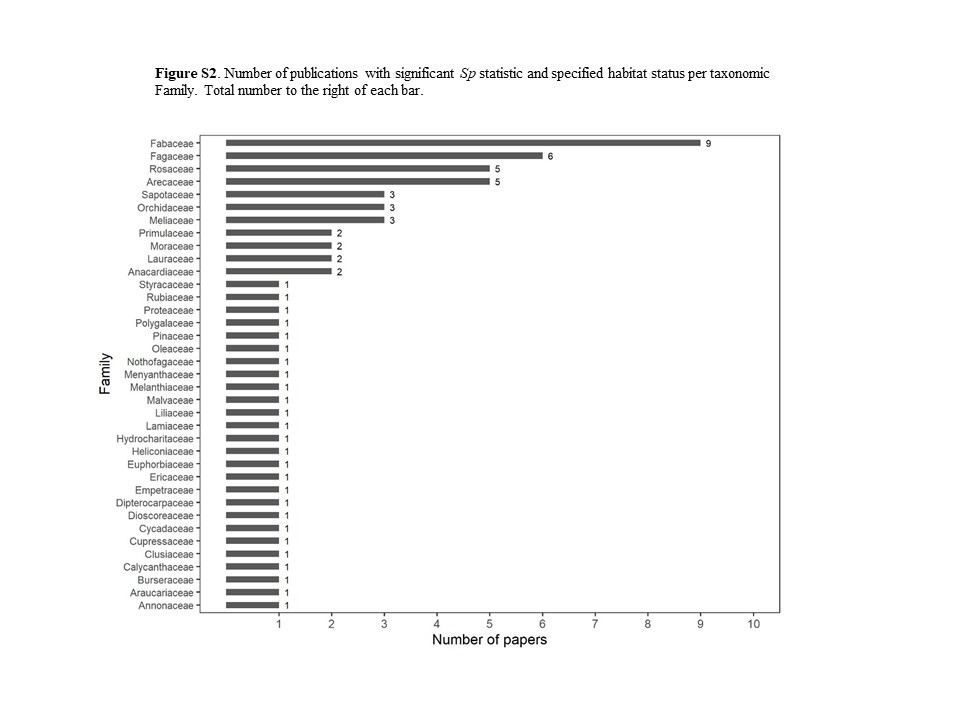

Supplement: plad019_suppl_Supplementary_Figure_S2 [file plad019_suppl_supplementary_figure_s2.jpeg]

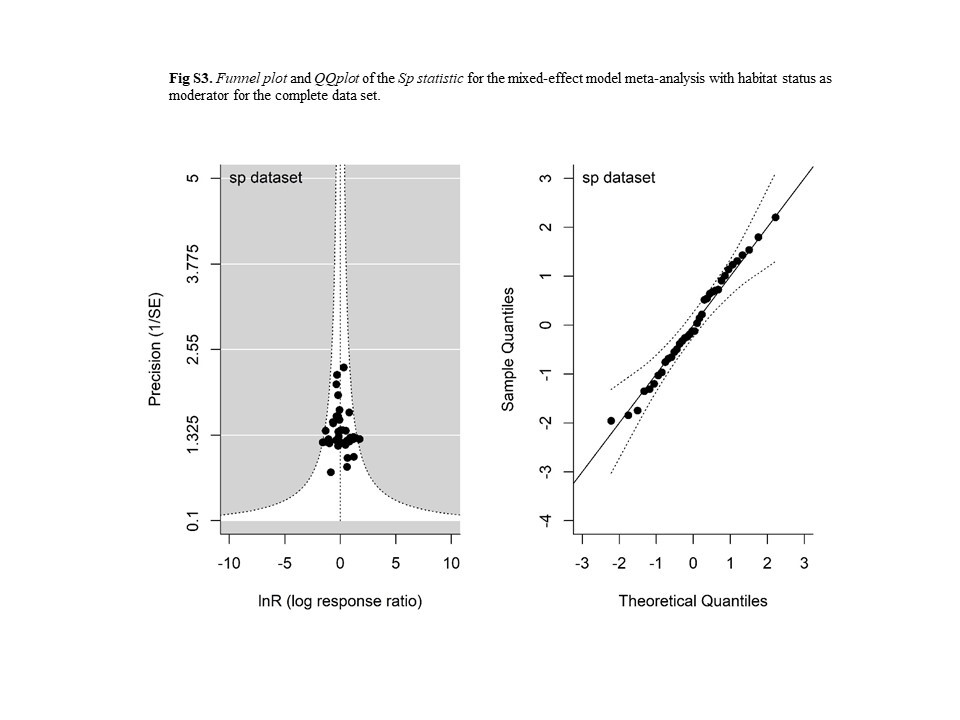

Supplement: plad019_suppl_Supplementary_Figure_S3 [file plad019_suppl_supplementary_figure_s3.jpeg]

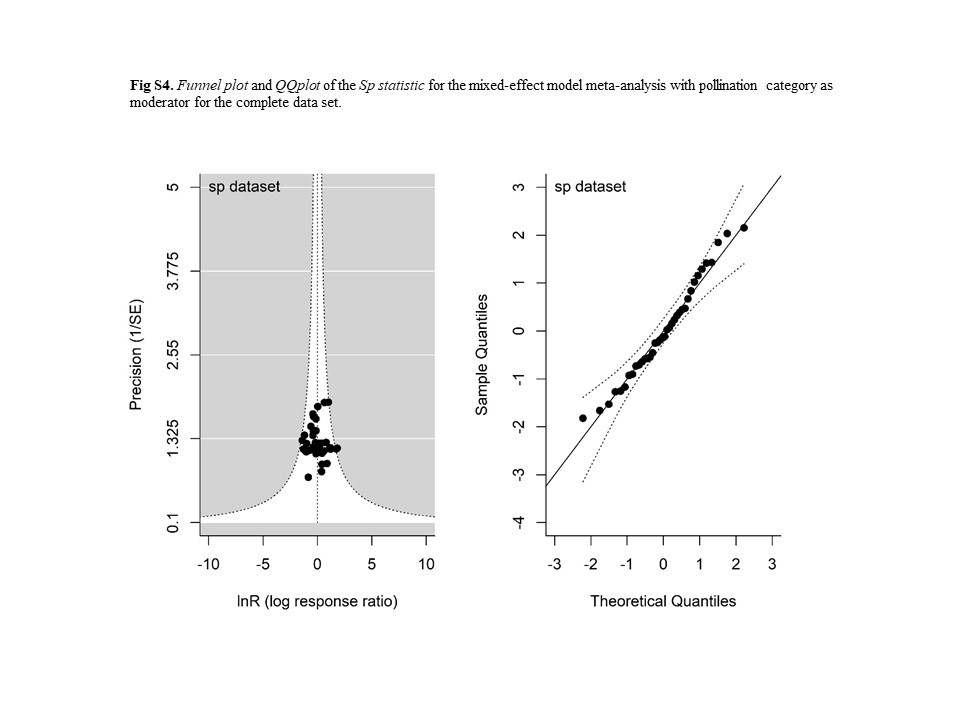

Supplement: plad019_suppl_Supplementary_Figure_S4 [file plad019_suppl_supplementary_figure_s4.jpeg]

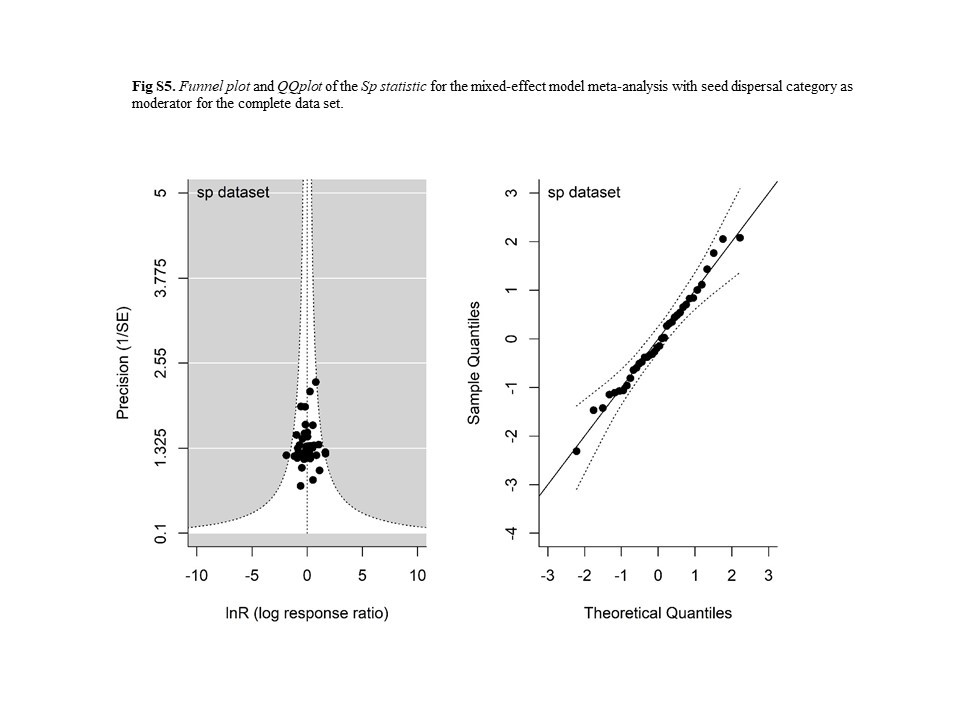

Supplement: plad019_suppl_Supplementary_Figure_S5 [file plad019_suppl_supplementary_figure_s5.jpeg]

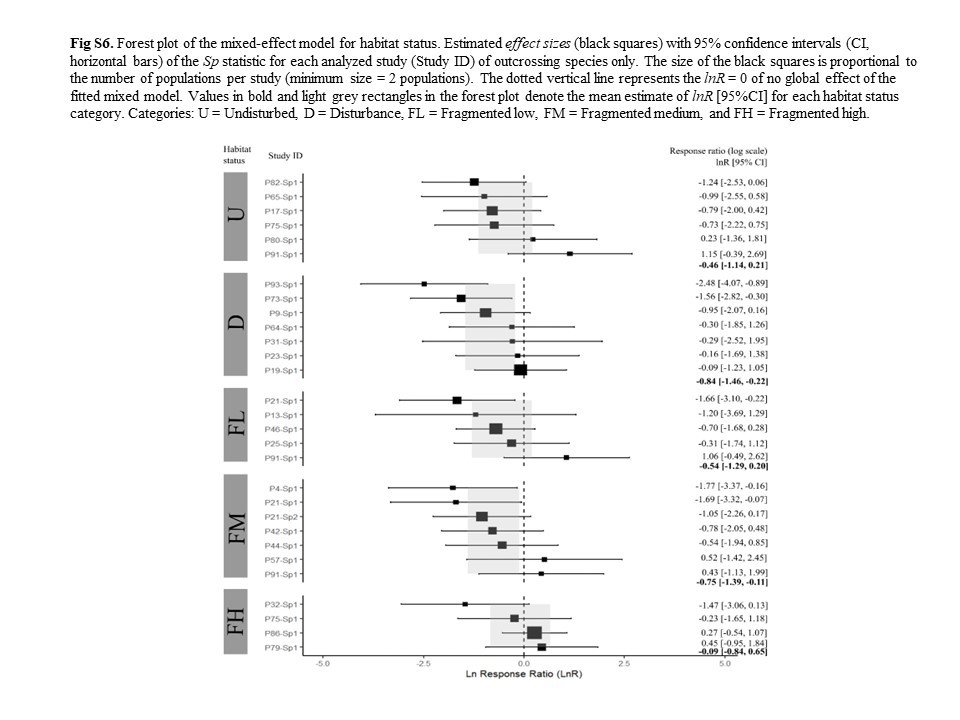

Supplement: plad019_suppl_Supplementary_Figure_S6 [file plad019_suppl_supplementary_figure_s6.jpeg]

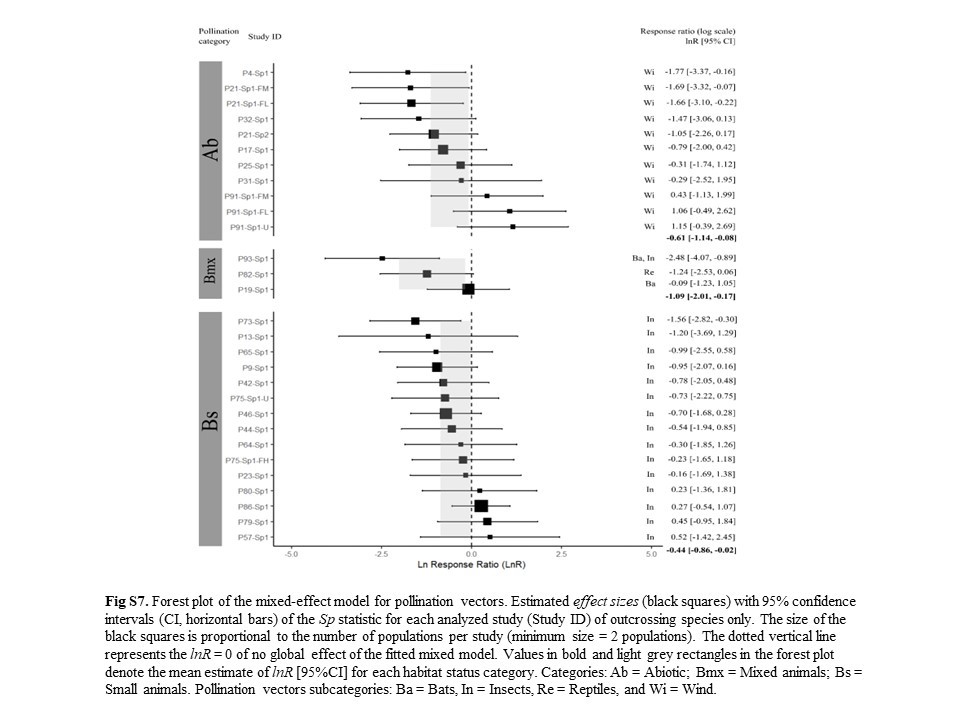

Supplement: plad019_suppl_Supplementary_Figure_S7 [file plad019_suppl_supplementary_figure_s7.jpeg]

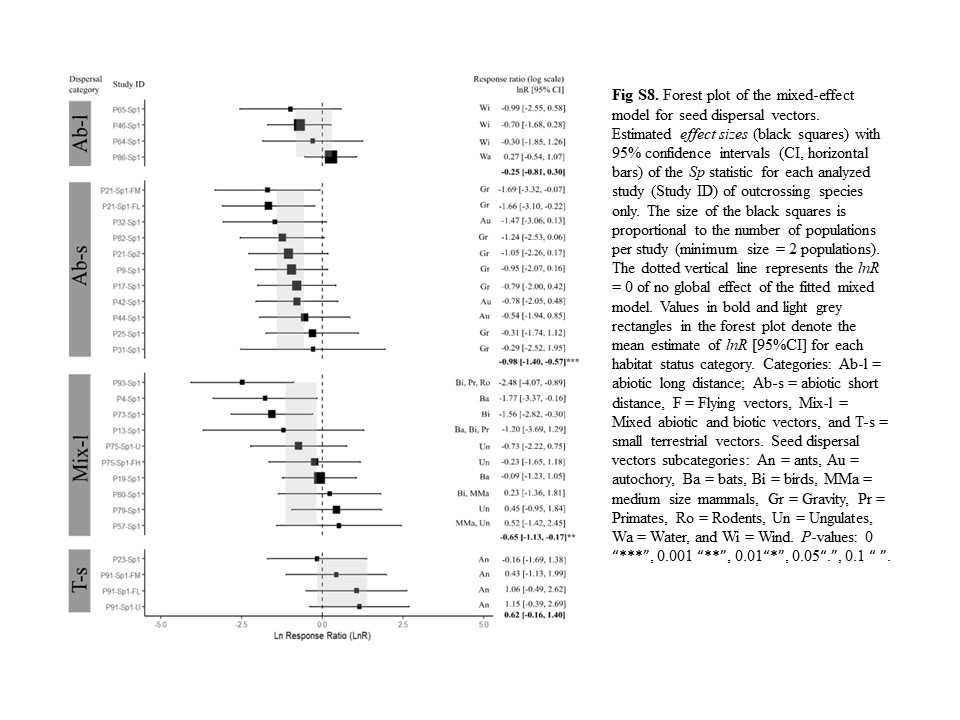

Supplement: plad019_suppl_Supplementary_Figure_S8 [file plad019_suppl_supplementary_figure_s8.jpeg]

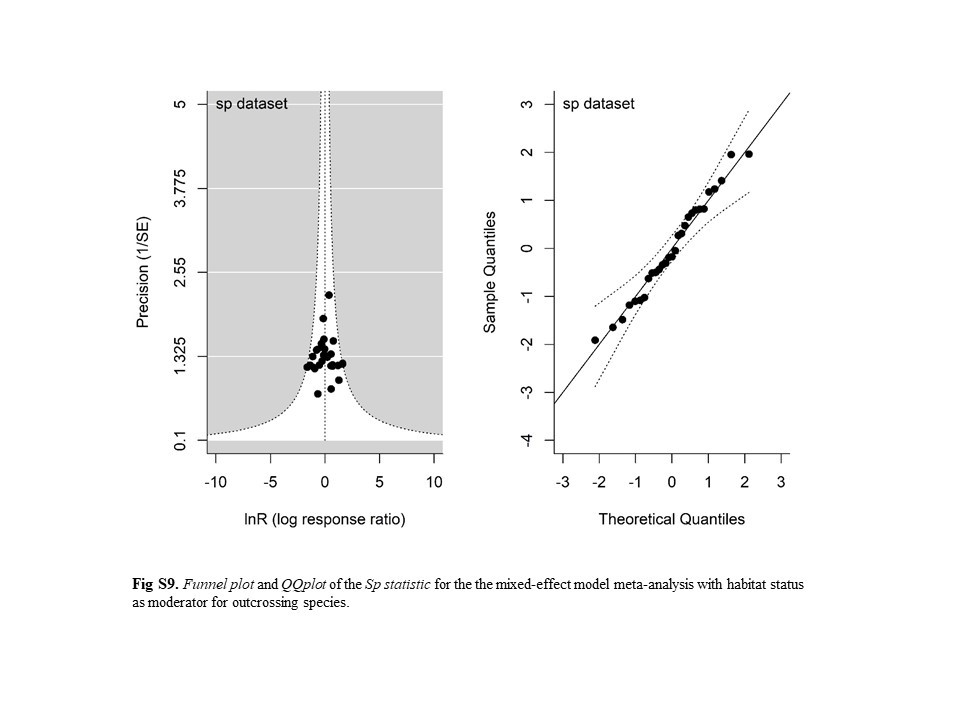

Supplement: plad019_suppl_Supplementary_Figure_S9 [file plad019_suppl_supplementary_figure_s9.jpeg]

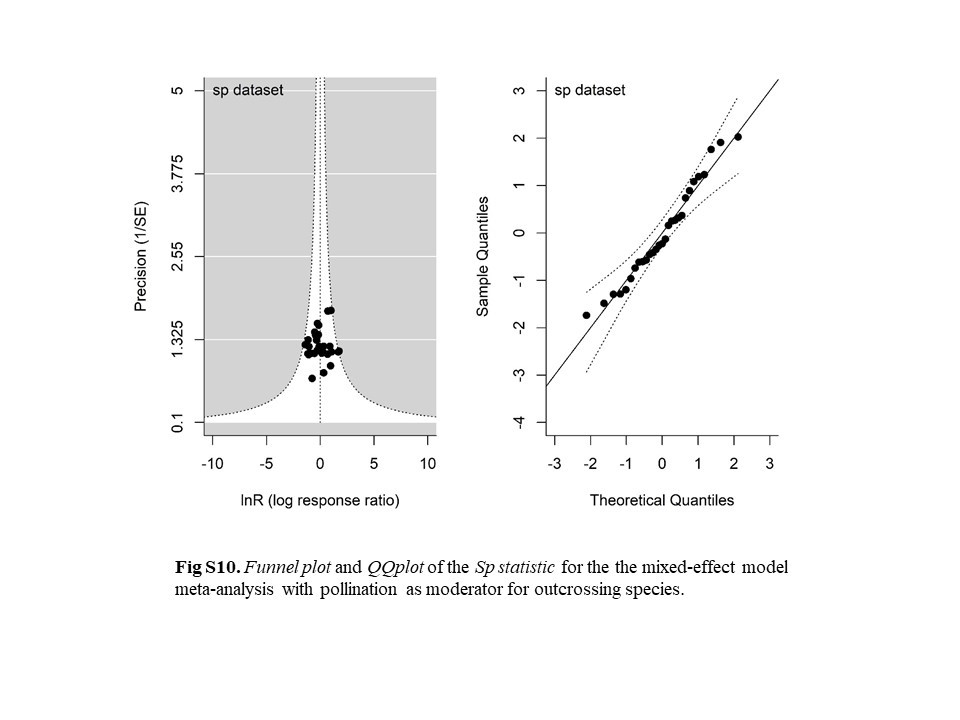

Supplement: plad019_suppl_Supplementary_Figure_S10 [file plad019_suppl_supplementary_figure_s10.jpeg]

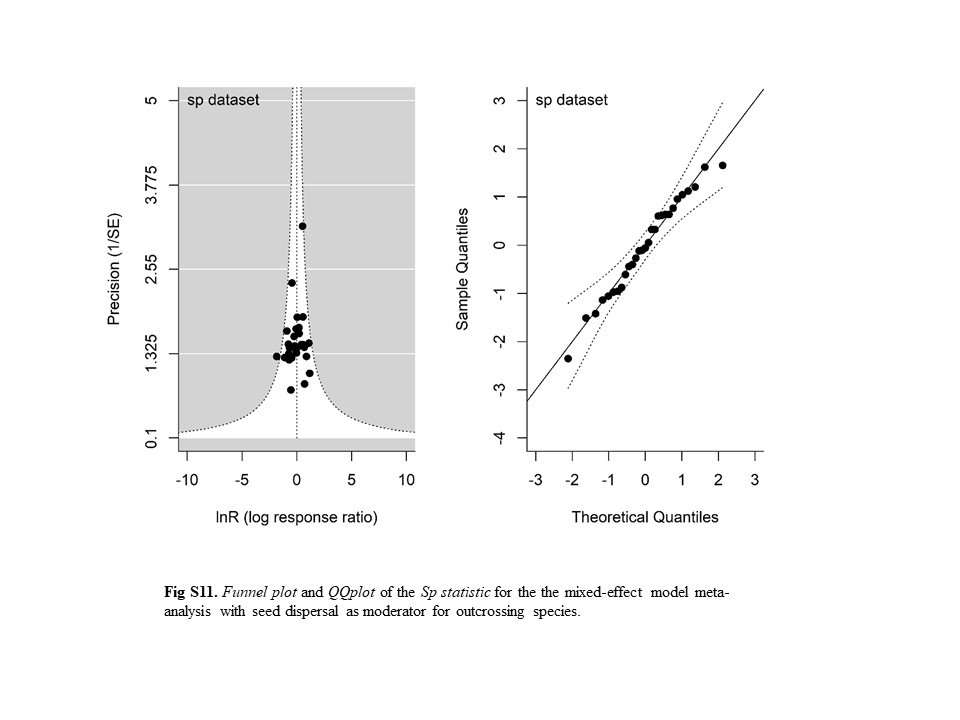

Supplement: plad019_suppl_Supplementary_Figure_S11 [file plad019_suppl_supplementary_figure_s11.jpeg]

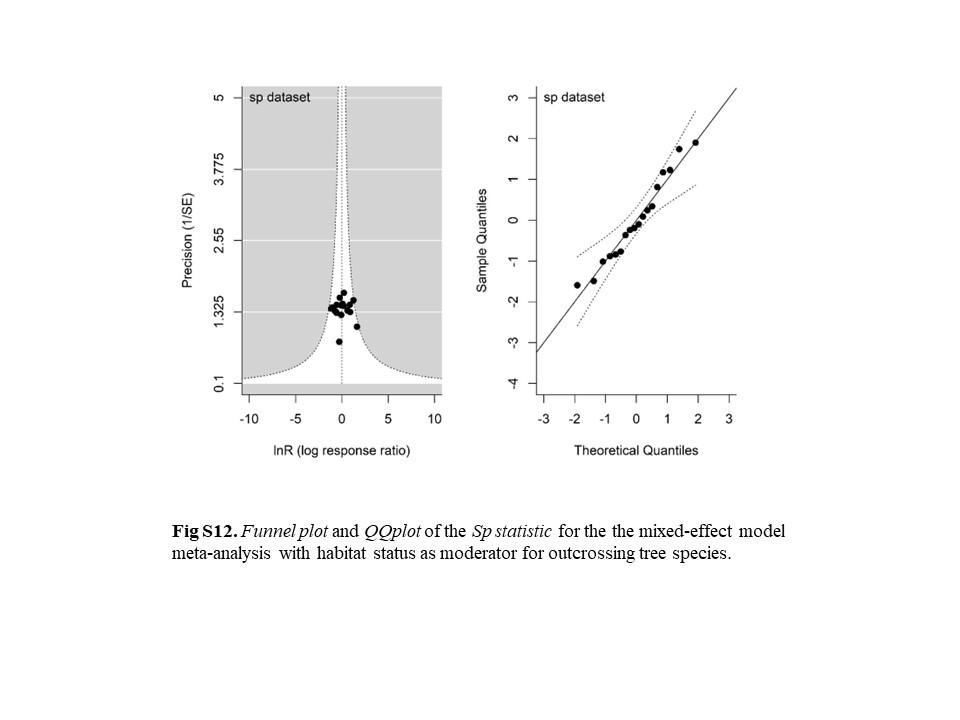

Supplement: plad019_suppl_Supplementary_Figure_S12 [file plad019_suppl_supplementary_figure_s12.jpeg]

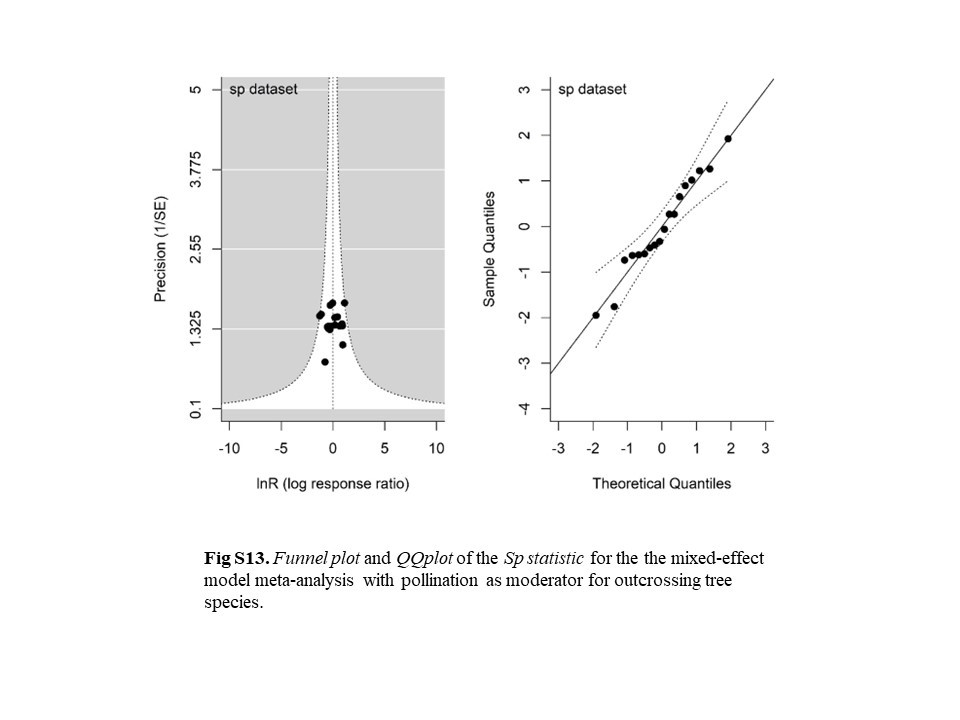

Supplement: plad019_suppl_Supplementary_Figure_S13 [file plad019_suppl_supplementary_figure_s13.jpeg]

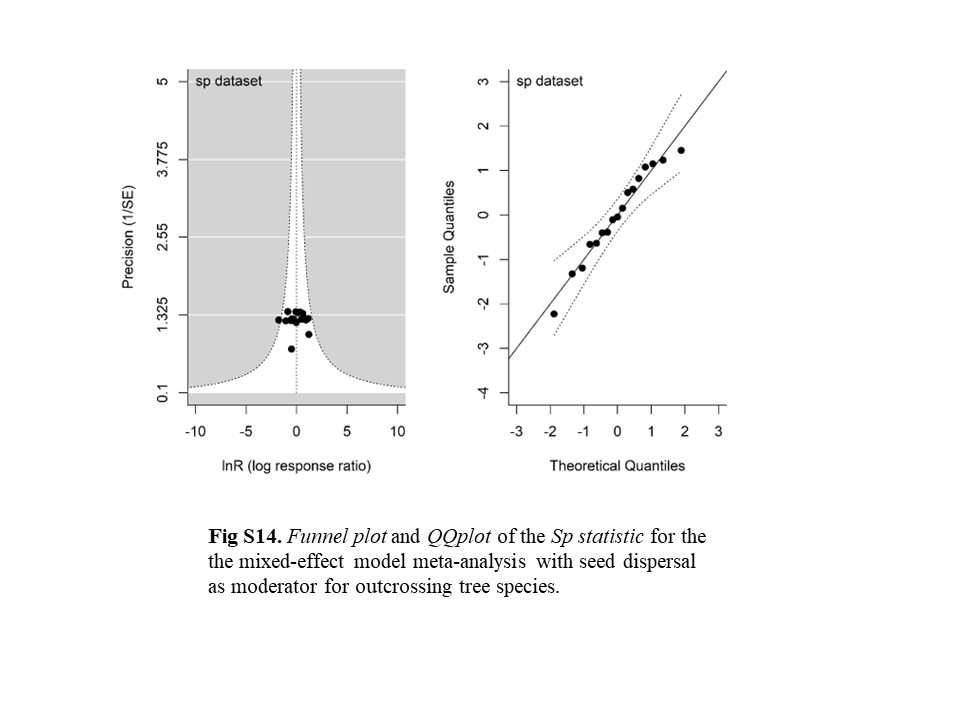

Supplement: plad019_suppl_Supplementary_Figure_S14 [file plad019_suppl_supplementary_figure_s14.jpeg]

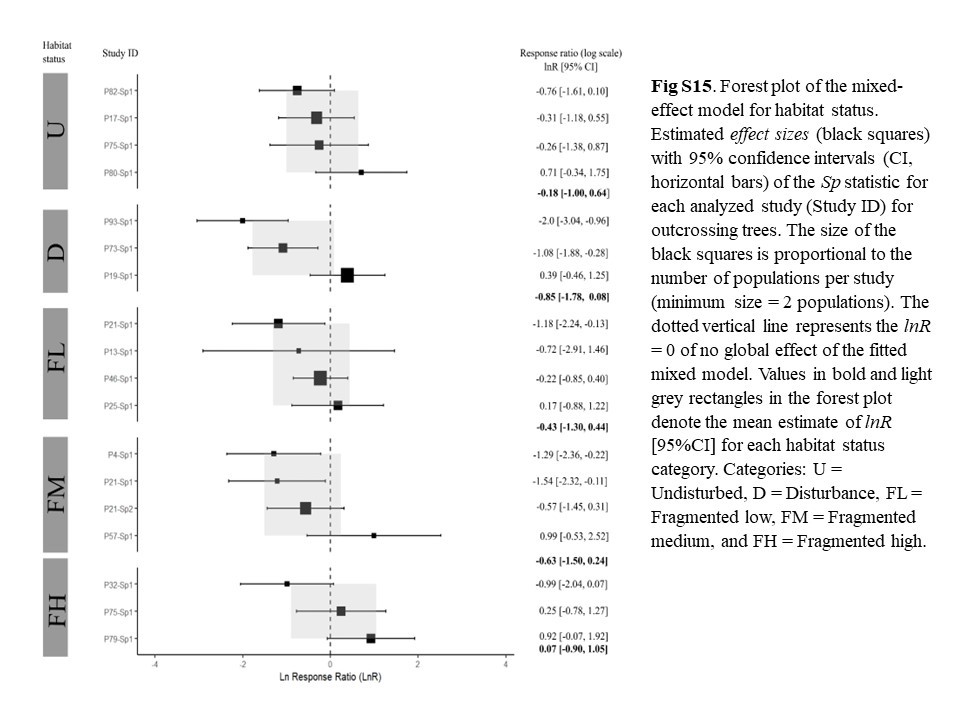

Supplement: plad019_suppl_Supplementary_Figure_S15 [file plad019_suppl_supplementary_figure_s15.jpeg]

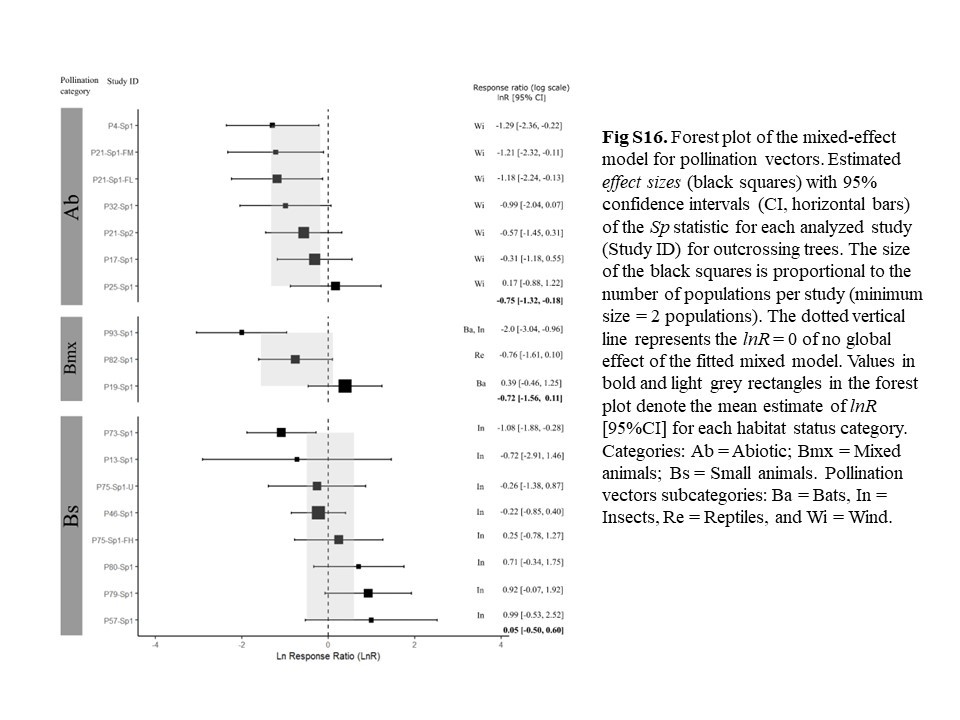

Supplement: plad019_suppl_Supplementary_Figure_S16 [file plad019_suppl_supplementary_figure_s16.jpeg]

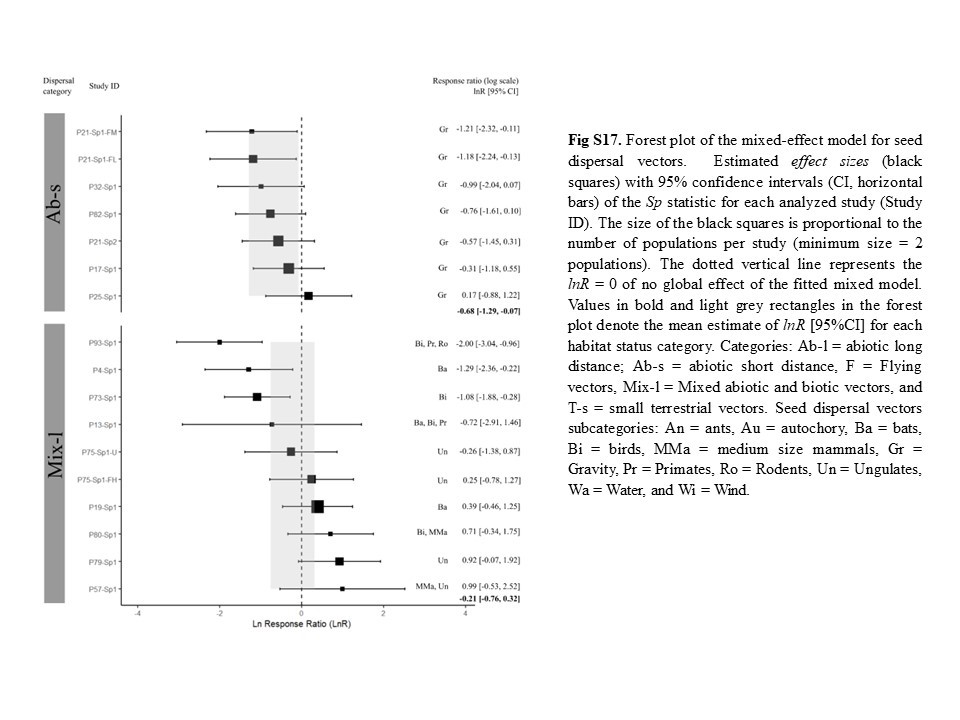

Supplement: plad019_suppl_Supplementary_Figure_S17 [file plad019_suppl_supplementary_figure_s17.jpeg]
