# Supplementary material for: Do habitat fragmentation and degradation influence the strength of fine-scale spatial genetic structure in plants? A global meta-analysis
Source: AoB Plants. 2023 May 3;15(3):plad019. doi: 10.1093/aobpla/plad019 (PMC10198778; doi:10.1093/aobpla/plad019)
Supplement: plad019_suppl_Supplementary_Table_S2 [file plad019_suppl_supplementary_table_s2.pdf]

Table S2. Statistics from meta-analyses with the complete, outcrossing and outcrossing tree species. *QE* refers to residual heterogeneity *QM* values for fitted model. Values in parenthesis denotes p-values. I^2 ratio of the total variance explained by the between-study variance, R^2 amount of accounted heterogeneity.

**Analysis**	**Moderators**	***dfE/dfM***	***QE* (*p value*)**	***QM (p value)***	**I^2 (%)**	**R^2 (%)**
Complete	Habitat status	33/4	42.723 (0.120)	5.964 (0.202)	17.22	39.05
	Pollination	35/2	46.977 (0.085)	2.862 (0.239)	25.06	3.93
	Seed dispersal	34/3	39.776 (0.228)	9.508 (0.023)	15.68	46.21
Outcrossing	Habitat status	24/4	33.715 (0.090)	2.743 (0.602)	25.52	1.67
	Pollination	26/2	36.534 (0.082)	1.626 (0.443)	29.04	0.00
	Seed dispersal	25/3	24.368 (0.498)	13.999 (0.003)	1.99	94.27
Tree and outcrossing	Habitat status	13/4	34.879 (0.001)	2.378 (0.666)	63.80	0.00
	Pollination	15/2	33.821 (0.004)	4.578 (0.101)	56.25	15.07
	Seed dispersal	15/1	38.393 (0.001)	1.234 (0.266)	61.58	0.91
