# Supplementary material for: Do habitat fragmentation and degradation influence the strength of fine-scale spatial genetic structure in plants? A global meta-analysis
Source: AoB Plants. 2023 May 3;15(3):plad019. doi: 10.1093/aobpla/plad019 (PMC10198778; doi:10.1093/aobpla/plad019)
Supplement: plad019_suppl_Supplementary_Table_S3 [file plad019_suppl_supplementary_table_s3.pdf]

Table S3. Statistics from ANOVA and Kruskal-Wallis tests for the *Sp* values in 177 plant populations under five habitat status categories. p-value between categories estimated with Tukey's test for ANOVA and Dunn test for Kruskal-Wallis, numbers in bold are statistically significant. Abbreviations: N = number of populations, U = Undisturbed, D = Disturbance, FL = Fragmented low, FM = Fragmented medium, and FH = Fragmented high.

Habitat Status				*p-value*	
Categories	N	Mean *Sp*		Tukey's test	Dunn test
U	39	0.024	U-D	0.353	1.000
D	40	0.014	U-FL	1.000	0.836
FL	24	0.024	U-FM	0.677	1.000
FM	32	0.017	U-FH	0.676	0.286
FH	42	0.030	D-FL	0.994	1.000
			D-FM	0.504	1.000
ANOVA *F (4,174) =3.137,* ***p=0.016***			D-FH	**0.014**	**0.008**
Kruskal-Wallis *H (4) =12.324,* ***p=0.015***			FM-FH	0.074	0.133
